# Genetic variants in purine metabolism and treatment resistance in schizophrenia: evidence supporting the adenosine hypothesis – a pilot study

**DOI:** 10.3389/fphar.2026.1876365

**Published:** 2026-07-28

**Authors:** Milica Pjevac, Tanja Blagus, Aleš Oblak, David Vogrinc, Tea Terzić, Jurij Bon, Vita Dolžan

**Affiliations:** 1 Centre for Clinical Psychiatry, University Psychiatric Clinic, Ljubljana, Slovenia; 2 Faculty of Medicine, University of Ljubljana, Ljubljana, Slovenia; 3 Pharmacogenetics Laboratory, Institute of Biochemistry and Molecular Genetics, Faculty of Medicine, University of Ljubljana, Ljubljana, Slovenia; 4 Department of Psychiatry, Faculty of Medicine, University of Ljubljana, Ljubljana, Slovenia

**Keywords:** adenosine hypothesis, AMPD1, ATIC, genetic polymorphism, ITPA, purine metabolism, schizophrenia, treatment-resistant schizophrenia

## Abstract

**Background:**

The adenosine hypothesis of schizophrenia provides an integrative framework in which reduced adenosinergic signalling contributes to dopaminergic hyperactivity and glutamatergic hypofunction. Enzymes regulating purine metabolism, including inosine triphosphatase (ITPA), adenosine monophosphate deaminase 1 (AMPD1), and 5-aminoimidazole-4-carboxamide ribonucleotide formyltransferase/IMP cyclohydrolase (ATIC), may influence adenosine availability and thereby modulate disease susceptibility and treatment response. As part of a pilot pharmacogenetic investigation, we investigated whether functional polymorphisms in these genes are associated with schizophrenia risk, treatment resistance, and symptom severity.

**Methods:**

A total of 249 patients with schizophrenia (including 47 with treatment-resistant schizophrenia - TRS) and 93 healthy controls were genotyped for *ITPA* rs1127354, *AMPD1* rs17602729, and *ATIC* rs2372536 in this exploratory, hypothesis-generating study. Case-control and TRS analyses were conducted using χ^2^ tests and logistic regression with Bonferroni correction. Symptom severity (BPRS), global functioning (GAF), and positive and negative symptom subscales were examined using two-way ANOVA with false discovery rate (FDR) adjustment for multiple exploratory comparisons.

**Results:**

*ITPA* rs1127354 showed a nominally significant recessive association with schizophrenia risk based on a very small genotype count (OR = 0.09, p = 0.033), driven by rare AA homozygosity, but did not remain significant after Bonferroni correction. No significant case-control associations were observed for AMPD1 or ATIC. In contrast, *ATIC* rs2372536 was significantly associated with TRS (χ^2^ = 10.86, p = 0.004; CG vs. CC OR = 2.68, 95% CI 1.20–6.55), remaining significant after Bonferroni correction. In exploratory dimensional analyses, uncorrected genotype-related effects were observed for global functioning and overall symptom severity; after FDR adjustment, only the association between *ATIC* rs2372536 and negative symptoms remained statistically supported at q < 0.10. *AMPD1* rs17602729 showed associations with overall symptom burden at the unadjusted level.

**Conclusion:**

Our findings provide preliminary evidence that genetic variation in purine metabolism may contribute to clinical heterogeneity in schizophrenia. In particular, *ATIC* rs2372536 was associated with treatment resistance and negative symptom severity, supporting a potential role of adenosinergic mechanisms in antipsychotic response. Associations with schizophrenia risk and other dimensions were exploratory and require replication in larger, independent cohorts. Integration of genetic data with biochemical measures of purine and adenosine metabolism will be important to establish clinical relevance and support precision-oriented therapeutic strategies.

## Introduction

Schizophrenia is a severe, chronic psychiatric disorder, with approximately 20%–30% of patients failing to achieve remission despite adequate antipsychotic treatment (Matrisciano, 2023). Its etiology is multifactorial, with a substantial but incompletely understood genetic component that interacts with neurodevelopmental, inflammatory, and other environmental factors. Dysregulation of dopaminergic and glutamatergic neurotransmission is central to symptom development, as supported by neuromorphological, molecular, pharmacological, and pharmacogenetic findings (McCutcheon et al., 2020). More recent work has also implicated additional neurotransmitter and neuroregulatory systems, neuroinflammatory processes, and metabolic pathways that converge on dopaminergic, glutamatergic, and serotonergic signalling in shaping vulnerability to psychosis (Trubalski et al., 2025). Within this framework, the adenosine hypothesis of schizophrenia provides an integrative model. Reduced adenosinergic signalling contributes to mesolimbic dopaminergic hyperactivity and cortical glutamatergic hypofunction. This may promote the emergence and persistence of psychotic symptoms (Boison et al., 2012). Adenosine is an endogenous purine nucleoside and neuromodulator involved in cellular bioenergetics, neuroimmune responses, and modulation of neurotransmission. These processes have all been implicated in the pathophysiology of schizophrenia. In line with this, reduced adenosine activity and alterations in adenosine receptors, transporters, and metabolic enzymes have been demonstrated in individuals with schizophrenia and in relevant animal models (Valle-León et al., 2021; Sahay et al., 2024).

Adenosine exerts its neuromodulatory effects through four G-protein-coupled receptors: A1 (ADORA1), A2A (ADORA2A), A2B (ADORA2B), and A3 (ADORA3), with A1 and A2A receptors playing a particularly important role in regulating dopaminergic and glutamatergic neurotransmission (Fredholm et al., 2011). Dysregulation of adenosine signalling, including altered receptor expression and function, has been linked to cognitive impairment and dopaminergic imbalance in schizophrenia (Cunha, 2016; O'Donovan et al., 2018). Consequently, pharmacological enhancement of adenosine signalling, especially via functional antagonism of D2 signalling via A2A receptors, is considered a promising therapeutic strategy, with potential benefits for negative and cognitive symptoms that remain insufficiently addressed by current antipsychotics (Yee et al., 2007).

Adenosine levels are tightly regulated by extracellular and intracellular enzymes that control its synthesis and metabolism. Adenosine kinase (ADK), which is predominantly expressed in astrocytes, phosphorylates adenosine to adenosine monophosphate (AMP), thereby promoting adenosine uptake from the extracellular space (Studer et al., 2006; Etherington et al., 2009). Experimental overexpression of ADK induces schizophrenia-like phenotypes in animal models, which can be ameliorated by ADK inhibition, although post-mortem studies have not demonstrated a simple direct proportional relationship between ADK activity and reduced adenosine levels in the frontal cortex of individuals with schizophrenia (Moody et al., 2020). These observations suggest that additional enzymes involved in purine turnover, including adenosine monophosphate deaminase 1 (AMPD1), inosine triphosphatase (ITPA), and 5-aminoimidazole-4-carboxamide ribonucleotide formyltransferase/IMP cyclohydrolase (ATIC), may also contribute to adenosinergic dysfunction (Frontera et al., 2021; Simone et al., 2013; Bulock et al., 2002).

AMPD1 catalyzes the conversion of AMP to inosine monophosphate (IMP); reduced or absent AMPD1 activity may therefore favor higher AMP and adenosine levels. The common *AMPD1* rs17602729 (C34T) variant introduces a premature stop codon and markedly reduces enzyme activity (Frontera et al., 2021). ITPA hydrolyses inosine triphosphate (ITP) to IMP, thereby maintaining the integrity of cellular nucleotide pools and indirectly influencing adenosine and purine homeostasis. The functional missense variant *ITPA* rs1127354 (94C>A; p.Pro32Thr) leads to pronounced loss of ITPA activity, with homozygotes typically showing near-complete enzyme deficiency, whereas heterozygotes exhibit substantially reduced activity (Sumi et al., 2002). ATIC is a bifunctional enzyme that catalyzes the final two steps of *de novo* purine synthesis and is functionally linked to folate metabolism. The missense variant *ATIC* rs2372536 (c.347C>G; p.Thr116Ser) has been associated with altered metabolism of 5-aminoimidazole-4-carboxamide ribonucleotide (AICAR) and reduced ATIC activity in pharmacogenetic studies, which may influence intracellular AICAR levels and downstream adenosine availability in specific contexts (Gallardo-Moya et al., 2025).

Pharmacotherapy for schizophrenia relies primarily on dopamine D2 receptor blockade to reduce mesolimbic hyperdopaminergia and alleviate positive symptoms. Given that A2A receptor activation functionally antagonizes D2 receptor signalling, interindividual variability in enzymes regulating adenosine availability could modulate both symptom expression and antipsychotic treatment response (Borroto-Escuela et al., 2020). Approximately one-third of patients develop treatment-resistant schizophrenia (TRS), defined by persistence of positive symptoms despite adequate treatment with at least two antipsychotics, and are typically candidates for clozapine, the gold standard treatment for TRS (Siskind et al., 2022). TRS appears to be associated with more severe symptomatology, earlier onset, poorer premorbid functioning, and distinct neurobiological features, but robust predictive biomarkers remain lacking.

Early identification of patients at increased risk of TRS is clinically important because delayed initiation of clozapine is associated with poorer outcomes and a lower likelihood of achieving remission (Potkin et al., 2020). While some demographic and clinical predictors of resistance have been proposed, no reliable biological markers are currently implemented in routine practice (Conley and Kelly, 2001). In this context, genetic variation in the adenosine pathway represents a plausible candidate for explaining interindividual differences in disease vulnerability, symptom profile, and antipsychotic response, especially given the central role of adenosine in coordinating dopaminergic and glutamatergic signalling. Consistent with the adenosinergic modulation hypothesis, allopurinol, a xanthine oxidase inhibitor that may enhance adenosinergic signalling, has been investigated as an adjunctive treatment in schizophrenia, with several randomized controlled trials suggesting modest improvement in clinical symptoms, although larger trials have reported mixed findings (Dickerson et al., 2009; Weiser et al., 2012).

Although large genome-wide association studies (GWAS) have not identified genome-wide significant signals at the ATIC, AMPD1, or ITPA loci in schizophrenia, this does not exclude context-dependent or pharmacogenetic effects of functional variants such as *ITPA* rs1127354, *ATIC* rs2372536, or *AMPD1* rs17602729 (Trubetskoy et al., 2022). Moreover, polymorphisms in adenosine receptor genes have been associated with symptom dimensions and with the severity of antipsychotic adverse effects, further supporting a role for adenosinergic mechanisms in clinical heterogeneity (Turčin et al., 2016). However, no study to date has specifically examined whether common functional polymorphisms in ATIC, AMPD1, and ITPA, which directly influence purine metabolism and adenosine homeostasis, are associated with schizophrenia risk, treatment resistance, or symptom severity.

The present study investigated whether common genetic variants in ATIC, AMPD1, and ITPA are associated with (i) the risk of schizophrenia, (ii) the development of TRS, and (iii) symptom severity in a Slovenian cohort of patients with schizophrenia. We hypothesized that genetic variation in purine metabolism may contribute to disease susceptibility, clinical heterogeneity, and variability in antipsychotic treatment response through its effects on adenosinergic signalling and its interaction with dopaminergic and glutamatergic neurotransmission.

## Materials and methods

### Participants and clinical data

A total of 249 patients with schizophrenia and 93 healthy controls were included. Patients were recruited from the University Psychiatric Clinic Ljubljana, and met DSM-IV criteria for schizophrenia, confirmed by structured clinical interviews conducted by psychiatrists. Exclusion criteria included poor treatment adherence, significant extrapyramidal symptoms during antipsychotic treatment (Abnormal Involuntary Movement Scale [AIMS] score ≥4 in any category), comorbid DSM-IV mental disorders, and major physical illnesses. Demographic and clinical data were extracted from medical records.

Of the 249 schizophrenia patients, 47 had TRS, and 202 were antipsychotic-responsive.

TRS was defined according to Conley and Kelly criteria, consistent with the Treatment Response and Resistance in Psychosis (TRRIP) consensus guidelines. It was operationalized as an inadequate response to two or more antipsychotics at adequate doses (chlorpromazine equivalents 400–600 mg/day) for 4–6 weeks, with persistent moderate-to-severe psychotic symptoms and functional decline lasting at least 5 years. Pseudoresistance was ruled out via systematic adherence assessment during hospitalization and outpatient follow-up.

Remission in the treatment-responsive group followed Andreasen and van Os criteria: mild or lesser severity (PANSS score ≤3) across eight items (P1, G2, P3, P2, G5, N1, N4, N6) representing negative, disorganization, and psychotic dimensions, sustained for ≥6 months.

Healthy controls were volunteers without a personal or family history of psychotic or major psychiatric disorders. Controls were substantially older (median 69.2 years, IQR 66.5–72.6) than cases (median 32.5 years, IQR 26–45) to minimize the risk of undiagnosed prodromal schizophrenia, although this introduces age-related confounding, as addressed in Limitations.

Participants were recruited and blood samples collected between 2010 and 2016. The study adhered to the Declaration of Helsinki and was approved by the National Medical Ethics Committee of the Republic of Slovenia (approvals KME 43/02/09, KME 0120-106/2024-2711-6, KME 44/12/16). All participants provided written informed consent.

### SNP selection

Three common functional single nucleotide polymorphisms (SNPs) in adenosine metabolic pathway genes *AMPD1* rs17602729 (p.Gln12Ter), *ATIC* rs2372536 (p.Thr116Ser), and *ITPA* rs1127354 (p.Pro32Thr) were selected based on: (1) known or predicted functional impact on enzyme activity; (2) sufficient minor allele frequency (MAF) > 5% in European populations to enable association testing: *ATIC* rs2372536 (c.347C>G; p.Thr116Ser; MAF ∼25% globally, 32% Europeans), *AMPD1* rs17602729 (c.34C>T; p.Gln12Ter; MAF 20%–30% Europeans), and *ITPA* rs1127354 (c.94C>A; p. Pro32Thr; MAF 7%–19%) (Karczewski et al., 2020) ; (3) prior evidence of associations with drug response or other phenotypes involving purine metabolism (Bulgay et al., 2024; Stocco et al., 2009; Marini et al., 2011).

### DNA isolation and genotyping

Genomic DNA was isolated from peripheral blood samples collected in EDTA-containing tubes using the FlexiGene DNA Kit (Qiagen, Hilden, Germany) or E.Z.N.A.® SQ Blood DNA Kit II (Omega Bio-tek, Inc., Norcross, GA, United States) according to the manufacturers’ instructions. DNA concentration was determined spectrophotometrically with Lambda BIO + UV/VIS (PerkinElmer, Massachusetts, United States). SNPs were genotyped using competitive allele-specific PCR with predesigned, validated “Kompetitive Allele-Specific PCR” (KASP™) assays (cat. no. KBS-2400-001; LGC Biosearch Technologies, Hoddesdon, UK) and KASP 2x Master Mix (cat. no. KBS-1016-002; LGC Biosearch Technologies) according to the manufacturer’s instructions. PCR was performed using a Veriti Thermal Cycler (Applied Biosystems, Thermo Fisher Scientific, Massachusetts, United States) or a ProFlex PCR System (Applied Biosystems). PCR thermal protocols were pre-optimised by the manufacturer; therefore, for ITPA and ATIC primers, a standard 61 °C–55 °C touchdown protocol was used, while PCR for the AMPD1 SNP was performed using a 2-step 57 °C protocol. The fluorescence intensity of the post-PCR products was measured using an Omega plate reader (BMG Labtech, Ortenberg, Germany) or a 7,500 Real-Time PCR System (Applied Biosystems). Genotype calls were analysed using the KlusterCaller analysis software (LGC Biosearch Technologies). As a quality control measure, 10% of the samples were genotyped in duplicate. All results were consistent.

### Statistical analysis

All statistical analyses were conducted in R and R Studio (Posit Team, 2025). Hardy–Weinberg equilibrium (HWE) was assessed in the control group using the genetics package (Bartlett, 2022).

Primary genetic association analyses tested differences in genotype distributions between (i) patients and controls and (ii) treatment-resistant versus treatment-responsive patients. Associations were tested under additive (χ^2^ test), dominant (Fisher’s exact test), and recessive models (pre-specified for functional variants). Logistic regression models were used as secondary analyses to estimate effect sizes (odds ratios and 95% confidence intervals) and to adjust for potential confounders. To account for multiple testing in primary analyses, Bonferroni correction was applied within each comparison (9 tests: 3 SNPs × 3 models = 9 tests for case – control and TRS), with an adjusted significance threshold of α_adj_ = 0.05/9 = 0.0056.

Secondary, exploratory analyses examined associations between genotype and clinical outcomes, including symptom severity (BPRS total, positive and negative subscales) and global functioning (GAF). Two-way ANOVA models (treatment-response group × genotype) were used, and partial eta squared (η^2^) was reported as a measure of effect sizes. Prior to analysis, extreme values were excluded according to pre-specified criteria. This resulted in reduced sample sizes in some genotype-by-group strata, particularly for less frequent genotypes, and ANOVA findings should therefore be interpreted with caution. To control for multiple testing in exploratory analyses, false discovery rate (FDR) correction was applied using the Benjamini–Hochberg procedure across 24 tests (3 SNPs × 4 outcomes × 2 effects [main and interaction]). Results were interpreted at both a conventional threshold (q < 0.05) and a more exploratory threshold (q < 0.10).

Given low-frequency genotypes, sensitivity analyses using Firth penalized logistic regression were performed to reduce small-sample bias.

All statistical tests were two-sided, and nominal significance was set at p < 0.05 unless otherwise specified.

## Results

### Sample characteristics

The study included 249 patients with schizophrenia (52.6% male; median age 32.5 years, IQR 26–45) and 93 healthy controls (48.4% male; median age 69.2 years, IQR 66.5–72.6). Median illness duration in patients was 9 years (IQR 6–14), and 46.6% were current smokers.

Among patients, 47 met criteria for TRS, and 202 were treatment-responsive. Compared with treatment-responsive patients, the TRS group was older (43.4 ± 11.1 vs. 41.4 ± 13.0 years), had longer illness duration (17.2 ± 9.5 vs. 9.0 ± 7.8 years), more hospitalizations (8.4 ± 7.6 vs. 2.9 ± 2.9), and higher symptom burden (BPRS total 41.0 ± 9.9 vs. 31.8 ± 12.2). Smoking prevalence was 45.7% in the TRS group and 58.9% in the treatment-responsive group.

Controls were older and had lower smoking prevalence (6.6% current smokers, 31.9% former smokers, and 61.5% non-smokers), reflecting intentional selection to minimize the likelihood of undiagnosed prodromal schizophrenia and ensure a stable population-based control sample.

### Genotype frequencies and schizophrenia risk

MAFs in controls were consistent with European reference populations and were: 11.8% for *AMPD1* rs17602729, 38.6% for *ATIC* rs2372536, and 12.0% for *ITPA* rs1127354. AMPD1 and ATIC were in Hardy-Weinberg equilibrium in controls, whereas ITPA rs1127354 deviated from HWE (exact test, p = 0.008), owing to an excess of AA homozygotes. As this same genotype underlies the nominal ITPA association in the case-control analysis, this finding is interpreted with caution as a possible genotyping artifact rather than a genuine protective effect (Table 1).

**TABLE 1:** Genotype frequencies, Minor Allele Frequencies (MAFs), and Hardy-Weinberg Equilibrium (HWE) in the control group.

Gene	SNP	Genotype	N (%)	MAF (%)	HWE p-value (controls)
*AMPD1*	rs17602729	CC	69 (77.5%)	11.80	0.807
​	​	CT	19 (21.3%)	​	​
​	​	TT	1 (1.1%)	​	​
*ATIC*	rs2372536	CC	38 (41.3%)	38.60	0.146
​	​	CG	37 (40.2%)	​	​
​	​	GG	17 (18.5%)	​	​
*ITPA*	rs1127354	CC	74 (80.4%)	11.96	0.008
​	​	CA	14 (15.2%)	​	​
​	​	AA	4 (4.3%)	​	​

Genotype counts differ due to missing data.

χ^2^ tests showed no significant case–control differences in genotype frequency distributions for *AMPD1* (χ^2^ = 0.68, p = 0.710) or *ATIC* (χ^2^ = 1.50, p = 0.473), as presented in Supplementary Table S1.

For *ITPA* rs1127354, genotype distributions differed nominally between patients and controls (χ^2^ = 7.01, p = 0.030), but this did not remain significant after Bonferroni correction (p_adj_ = 0.27). AA homozygotes were less frequent among patients than controls, suggesting a nominal recessive protective effect (recessive model: OR = 0.09, 95% CI 0.01–0.83, p = 0.033 uncorrected; additive trend p = 0.078). However, this estimate is highly unstable, and the wide confidence interval reflects sparse data (Table 2). Post-hoc power analysis indicated limited power (∼25%) to detect recessive effects for *ITPA* rs1127354 at α = 0.05.

**TABLE 2:** Frequency distribution of the investigated genotypes and risk for schizophrenia (univariate logistic regression analysis).

SNP	Genotype	Control group N (%)	Patient group N (%)	OR (95% CI)	p-value
*AMPD1* rs17602729	CC	69 (77.5)	184 (73.9)	Reference	​
​	CT	19 (21.3)	63 (25.3)	1.24 (0.69–2.23)	0.464
​	TT	1 (1.0)	2 (0.8)	0.75 (0.07–8.40)	0.816
*ATIC* rs2372536	CC	38 (41.3)	89 (38.7)	Reference	​
​	CG	37 (40.2)	108 (47.0)	1.25 (0.73–2.12)	0.418
​	GG	17 (18.5)	33 (14.3)	0.83 (0.41–1.67)	0.598
*ITPA* rs1127354	CC	74 (80.4)	203 (85.7)	Reference	​
​	CA	14 (15.2)	33 (13.9)	0.86 (0.44–1.70)	0.662
​	AA	4 (4.3)	1 (0.4)	0.09 (0.01–0.83)	**0.033** *

* p < 0.05 indicates nominal statistical significance; genotype counts differ due to missing data (for AMPD1 controls 4; ATIC, controls 1, patient group 19; ITPA, controls 1; patient group 12).

Multivariate adjustment (age/sex/smoking) was unstable in the rare AA stratum; unadjusted primary results were reported cautiously, consistent with pilot findings.

### Genotype associations with treatment resistance

Genotype distributions of *AMPD1* rs17602729, *ATIC* rs2372536, and *ITPA* rs1127354 in treatment-resistant versus treatment-responsive patients are summarized in Supplementary Table S2.

A significant difference between groups was observed for *ATIC* rs2372536 in the primary χ^2^ analysis (χ^2^ = 10.86, p = 0.004), remaining below the Bonferroni-adjusted threshold (α_adj = 0.0056), as presented in Supplementary Table S3. The effective sample size for this comparison was reduced to 35 TRS patients with available ATIC genotypes. *AMPD1* rs17602729 and *ITPA* rs1127354 showed no significant associations (p = 0.526 and p = 0.864, respectively).

Logistic regression analysis, performed as a secondary analysis, yielded consistent effect estimates, with CG carriers showing increased odds of treatment resistance compared with CC carriers (OR = 2.68, 95% CI 1.20–6.55, p = 0.019). Estimates for the GG genotype were not reliable due to sparse data.

In multivariable models (Table 3) adjusted for sex and smoking, the association remained significant (adjusted OR = 2.79, 95% CI 1.20–6.50, p = 0.017), with similar results after additional adjustment for age (adjusted OR = 2.80, 95% CI 1.20–6.55, p = 0.017). The consistency of effect estimates between unadjusted and adjusted models suggests that the observed association is unlikely to be driven by major demographic confounders.

**TABLE 3:** Logistic regression analyses of AMPD1 rs17602729, ATIC rs2372536, and ITPA rs1127354 with treatment-resistant schizophrenia (TRS).

Gene/SNP	Model	n	TRS n	Unadjusted OR (95% CI), p	Adjusted (sex, smoke) OR (95% CI), p	Adjusted (sex, smoke, age) OR (95% CI), p
*AMPD1* rs17602729	Additive	243	46	1.17 (0.60–2.30) p = 0.643	1.26 (0.64–2.51) p = 0.504	1.26 (0.63–2.50) p = 0.508
​	Dominant	243	46	1.10 (0.54–2.25) p = 0.797	1.17 (0.57–2.43) p = 0.667	1.18 (0.57–2.44) p = 0.659
​	Recessive	243	46	4.36 (0.27–70.96) p = 0.301	6.12 (0.36–104.37) p = 0.211	5.40 (0.31–94.12) p = 0.247
​	CT vs. CC	243	46	1.01 (0.49–2.10) p = 0.978	1.06 (0.51–2.24) p = 0.868	1.08 (0.51–2.26) p = 0.849
*ATIC* rs2372536	Additive	224	34	1.04 (0.61–1.76) p = 0.892	1.05 (0.61–1.81) p = 0.863	1.07 (0.62–1.86) p = 0.800
​	Dominant	224	34	1.98 (0.88–4.47) p = 0.101	1.95 (0.85–4.45) p = 0.114	2.01 (0.87–4.61) p = 0.102
​	Recessive	224	34	0.16 (0.02–1.18) p = 0.072	0.17 (0.02–1.28) p = 0.084	0.17 (0.02–1.33) p = 0.091
​	CG vs. CC	230	35	**2.68 (1.20–6.55) p = 0.019** *	**2.79 (1.20–6.50) p = 0.017** *	**2.80 (1.20–6.55) p = 0.017** *
*ITPA* rs1127354	Additive	232	39	0.84 (0.31–2.26) p = 0.731	0.87 (0.32–2.32) p = 0.774	0.81 (0.30–2.20) p = 0.682
​	Dominant	232	39	0.87 (0.31–2.40) p = 0.783	0.91 (0.32–2.58) p = 0.866	0.85 (0.30–2.43) p = 0.766
​	CA vs. CC	232	39	0.90 (0.33–2.52) p = 0.847	0.98 (0.35–2.78) p = 0.975	0.92 (0.32–2.62) p = 0.873

OR, odds ratio; CI, confidence interval. Reference genotype: major homozygote (CC) for all SNPs. Additive model: minor allele count (0/1/2). Dominant: any minor allele vs. reference homozygote. Recessive: minor homozygote vs. rest. Het vs. ref (CT/CG/CA, vs. CC): heterozygote vs. reference. For ATIC, rs2372536 CG, vs. CC, values are from the primary analysis (n = 35 TRS, with available ATIC, genotype). All other rows calculated from the complete available dataset. Adjusted models: Model 1 includes sex (0 = male, 1 = female) and smoking (0 = non-smoker, 1 = smoker); Model 2 additionally includes age. Bonferroni-corrected significance threshold α = 0.0056 (9 tests: 3 SNPs × 3 genetic models). ITPA, recessive model omitted (no TRS, patients with AA genotype; OR, not estimable).

* p < 0.05 (nominal). Bold: p < 0.0056 (Bonferroni-corrected).

Sensitivity analysis using Firth-penalized logistic regression to account for sparse genotype counts yielded comparable effect estimates, supporting the robustness of the association. Post hoc power analysis indicated approximately 70% power to detect the observed effect (OR = 2.68) at α_adj = 0.0056.

No significant associations with treatment resistance were observed for *AMPD1* rs17602729 or *ITPA* rs1127354, consistent with χ^2^ analyses.

### Associations of genotype and treatment-response group with symptom severity and functioning

Two-way ANOVA analyses were performed to examine the effects of treatment-response group and genotypes on clinical outcomes; the results are presented in Table 4.

**TABLE 4:** Two-way ANOVA results for AMPD1, ATIC, and ITPA genotypes across clinical outcomes.

Outcome	SNP	Group effect	Genotype effect	Interaction
BPRS total	*AMPD1* rs17602729	F (1,113) = 2.044, p = 0.156	F (1,113) = 4.997, p = 0.027, η^2^ = 0.042	F (1,113) = 0.206, p = 0.651
​	*ATIC* rs2372536	F (1,97) = 1.301, p = 0.257	F (2,97) = 0.200, p = 0.819	F (1,97) = 6.577, p = 0.012, η^2^ = 0.062
​	*ITPA* rs1127354	F (1,100) = 1.949, p = 0.166	F (1,100) = 0.145, p = 0.704	F (1,100) = 0.003, p = 0.955
GAF	*AMPD1* rs17602729	F (1,91) = 47.253, p < 0.001, η^2^ = 0.339	F (1,91) = 0.836, p = 0.363	F (1,91) = 0.167, p = 0.683
​	*ATIC* rs2372536	F (1,92) = 50.202, p < 0.001, η^2^ = 0.327	F (2,92) = 4.376, p = 0.015, η^2^ = 0.057	F (1,92) = 2.513, p = 0.116
​	*ITPA* rs1127354	F (1,80) = 59.088, p < 0.001, η^2^ = 0.421	F (1,80) = 0.964, p = 0.329	F (1,80) = 0.377, p = 0.541
Positive symptoms (P_sum)	*AMPD1* rs17602729	F (1,91) = 46.258, p < 0.001, η^2^ = 0.332	F (1,91) = 0.482, p = 0.489	F (1,91) = 1.795, p = 0.184
​	*ATIC* rs2372536	F (1,92) = 50.125, p < 0.001, η^2^ = 0.334	F (2,92) = 2.691, p = 0.073	F (1,92) = 2.734, p = 0.102
​	*ITPA* rs1127354	F (1,80) = 56.945, p < 0.001, η^2^ = 0.414	F (1,80) = 0.249, p = 0.619	F (1,80) = 0.227, p = 0.635
Negative symptoms (N_sum)	*AMPD1* rs17602729	F (1,91) = 33.815, p < 0.001, η^2^ = 0.270	F (1,91) = 0.397, p = 0.530	F (1,91) = 0.140, p = 0.709
​	*ATIC* rs2372536	F (1,92) = 38.813, p < 0.001, η^2^ = 0.266	F (2,92) = 6.803, p = 0.002, η^2^ = 0.093	F (1,92) = 1.344, p = 0.249
​	*ITPA* rs1127354	F (1,80) = 48.492, p < 0.001, η^2^ = 0.371	F (1,80) = 1.872, p = 0.175	F (1,80) = 0.380, p = 0.539

Abbreviations: BPRS, brief psychiatric rating scale; GAF, global assessment of functioning; P_sum, positive symptom subscale; N_sum, negative symptom subscale; TRS, treatment-resistant schizophrenia; partial η^2^, partial eta squared (effect size). Nominal p-values are presented. False discovery rate (FDR)–adjusted results are described in the text. Only the ATIC, rs2372536 main effect genotype on negative symptom severity (N_sum) survived FDR, correction at q < 0.10. Note: Two-way ANOVA, was conducted with group (treatment-responsive vs. treatment-resistant) and genotype as fixed factors. Effect sizes (partial η^2^) are reported for genotype and interaction effects. For SNPs, with only two effective genotype categories in the analytical sample, following outlier exclusion (AMPD1 rs17602729 and ITPA, rs1127354), all model terms were estimated with df = 1. For ATIC, rs2372536, the genotype main effect was estimated with df = 2 (three genotype groups present); however, the interaction term was estimated with df = 1 due to a single GG, homozygote in the TRS, stratum, rendering this cell statistically unreliable. Results involving sparse cells should be interpreted with caution.

Significant main effects of treatment-response group were observed for global functioning (GAF) and positive and negative symptom scores (all p < 0.001), with treatment-resistant patients exhibiting poorer functioning and greater symptom burden. In contrast, no significant group effect was observed for overall symptom severity (BPRS total).

A nominal main effect of *AMPD1* rs17602729 genotype on BPRS total score was observed (F (1,113) = 4.997, p = 0.027, partial η^2^ = 0.042), indicating differences in overall psychopathology across AMPD1 genotype groups. No significant group effect or group × genotype interaction was detected for this SNP, and this association did not remain significant after correction for multiple testing.

For *ATIC* rs2372536, a group × genotype interaction effect on BPRS total score (F (1,97) = 6.577, p = 0.012, partial η^2^ = 0.062) was observed, suggesting that the relationship between ATIC genotype and overall symptom severity differed between treatment-resistant and treatment-responsive patients. In addition, nominal main effects of *ATIC* genotype were detected on global functioning (GAF) (F (2,92) = 4.376, p = 0.015, partial η^2^ = 0.057) and negative symptom severity (N_sum; F (2,92) = 6.803, p = 0.002, partial η^2^ = 0.093). After applying Benjamini–Hochberg false discovery rate (FDR) correction across 24 exploratory tests, only the association between *ATIC* rs2372536 and negative symptom severity remained significant at q < 0.10, whereas no associations remained significant at q < 0.05. Overall, these findings should be considered exploratory, particularly in light of reduced sample sizes and sparse genotype-by-group cells following outlier exclusion.

For *ITPA* rs1127354, no significant main or interaction effects were observed for any clinical outcome. Given reduced sample sizes in some genotype-by-group strata following outlier exclusion, these findings should be interpreted with caution and considered exploratory.

## Discussion

### Key findings

To our knowledge, this is the first study to examine functional polymorphisms in ITPA, ATIC, and AMPD1, key regulators of purine metabolism and adenosine homeostasis in relation to schizophrenia risk and TRS. In our Slovenian cohort, no robust associations were observed between the investigated genetic variants in the adenosine pathway and schizophrenia risk after correction for multiple testing. A nominal recessive association was identified for *ITPA* rs1127354, driven by a lower frequency of AA homozygotes among patients; however, this finding did not withstand correction and was based on very small genotype counts, and should therefore be interpreted with caution.

In contrast, *ATIC* rs2372536 showed a significant association with TRS, with the CG genotype associated with increased odds of treatment resistance. This association remained consistent after adjustment for sex, age, and smoking status, and was supported by sensitivity analyses.

In exploratory, hypothesis-generating analyses of clinical dimensions, nominal genotype-related effects were observed for overall symptom severity, global functioning, and negative symptoms. These findings should be interpreted with caution, as they were not the primary aim of this study and were conducted in reduced analytical samples following outlier exclusion. After FDR adjustment, only the association between *ATIC* rs2372536 and negative symptoms remained statistically supported at q < 0.10; no associations survived the conventional threshold of q < 0.05.

Overall, our pilot findings provide preliminary evidence linking *ATIC* rs2372536 to treatment resistance — a Bonferroni-corrected primary finding supported by ∼70% statistical power — and, at an exploratory level, to negative symptom expression. In contrast, findings for *ITPA* rs1127354, *AMPD1* rs17602729, and all dimensional analyses should be considered hypothesis-generating only, requiring replication in larger, adequately powered cohorts before any conclusions can be drawn.

### *ITPA* rs1127354 and schizophrenia risk

*ITPA* rs1127354 (p.Pro32Thr) is a well-characterized loss-of-function variant associated with markedly reduced enzyme activity. Although absent from schizophrenia GWAS hits, it has been established as pharmacogenetically relevant in thiopurine and ribavirin metabolism (Marsh et al., 2004). Given the established functional effects, recessive models were specified “*a priori*”. The *ITPA* rs1127354 variant showed a nominal recessive association with schizophrenia diagnosis, driven by a lower frequency of AA homozygotes among cases compared with controls. However, this observation did not survive correction for multiple testing and was based on very small genotype counts, resulting in a highly unstable effect estimate with a wide confidence interval.

Given the limited statistical power and lack of support from genome-wide association studies, this finding should be considered exploratory. However, given the established functional impact of the rs1127354 variant on ITPA enzyme activity, further investigation in larger, adequately powered cohorts would be required to determine whether this signal reflects a true protective association or a sampling artifact.

### *ATIC* rs2372536 and treatment-resistant schizophrenia

*ATIC* rs2372536 emerged as the most consistent genetic signal in this study. Hardy–Weinberg equilibrium was assessed in controls, as recommended for genetic association studies. In the TRS group, the observed genotype distribution for *ATIC* rs2372536 showed a marked underrepresentation of GG homozygotes, consistent with the direction of the association. Given the small subgroup size, this pattern is most likely attributable to sampling variability rather than a deviation requiring formal interpretation. The observed pattern suggests that variation in *ATIC* may contribute to susceptibility to treatment resistance, with effects that were consistent across analytical approaches. In addition, exploratory findings suggest that this variant may influence negative symptom severity and global functioning, indicating that its effects could extend beyond categorical treatment response to broader dimensions of clinical heterogeneity. Although only the association with negative symptoms remained statistically supported after correction for multiple testing, this pattern is consistent with a potential contribution of ATIC variation to antipsychotic response heterogeneity, particularly in relation to negative symptom expression. Functionally, ATIC catalyses the final steps of *de novo* purine biosynthesis and is linked to folate-dependent one-carbon metabolism (Wizrah et al., 2022). The rs2372536 missense variant has been associated with altered enzymatic efficiency and may influence intracellular AICAR levels and downstream adenosine availability (Borroto-Escuela et al., 2020; Wizrah et al., 2022). Within the framework of the adenosine hypothesis, altered adenosinergic signalling could modulate A2A receptor–mediated inhibition of dopaminergic D2 signalling, thereby potentially influencing antipsychotic responsiveness and negative symptom expression.

Taken together, these findings support the hypothesis that variation in purine metabolism may contribute to interindividual differences in antipsychotic treatment response, potentially through modulation of A2A–D2 receptor interactions within the adenosine hypothesis framework. However, these mechanistic interpretations remain speculative and require confirmation in studies integrating genetic and biochemical measures of purine metabolism.

### Dimensional symptom analyses

Exploratory analyses of dimensional clinical outcomes indicated nominal genotype-related differences across selected measures. Nominal associations were observed between *ATIC* rs2372536 and negative symptom severity, global functioning, and the interaction with overall symptom burden, as well as between *AMPD1* rs17602729 and total BPRS score. However, after controlling for the false discovery rate across multiple exploratory comparisons, only the association between *ATIC* rs2372536 and negative symptom severity remained statistically supported at a liberal FDR threshold.

Overall, these findings should be considered hypothesis-generating and require replication in larger cohorts.

### Therapeutic implications

The observed association between *ATIC* rs2372536 and treatment resistance may have implications for pharmacogenetic stratification. Given the role of adenosine in modulating dopaminergic signalling through A2A–D2 receptor interactions, interindividual variability in purine metabolism may contribute to differences in antipsychotic response, particularly in patients with persistent positive or prominent negative symptoms.

Adjunctive strategies targeting purine metabolism have already been explored in schizophrenia. Several smaller randomized controlled trials have reported modest symptomatic improvement with the xanthine oxidase inhibitor allopurinol when added to antipsychotic treatment (Akhondzadeh et al., 2005; Brunstein et al., 2005). However, larger and more methodologically rigorous trials have yielded mixed or non-significant results (Hirota and Kishi, 2013). These inconsistencies may reflect the marked biological heterogeneity of schizophrenia rather than the absence of a mechanistic effect. One potential explanation for these mixed findings is biological heterogeneity across patient populations. Genetic stratification based on variants affecting adenosine, including *ATIC* rs2372536, may help identify subgroups more likely to benefit from such interventions.

An important interpretive consideration is whether the observed association reflects a pharmacogenetic effect on antipsychotic response rather than underlying disease biology. Antipsychotic medications can influence purinergic signalling and adenosine receptor activity, particularly through A2A–D2 receptor interactions (Lara et al., 2006; Ferré et al., 1997), and genetic variation in ATIC may modulate these effects. The present design does not allow distinction between pharmacodynamic and disease-related mechanisms, which should be addressed in future prospective studies. While the present study does not directly assess response to adenosine-modulating agents, the findings support further investigation of purine pathway genetics in conjunction with biochemical measures of adenosine metabolism and treatment response data in prospective, biomarker-driven studies.

## Study limitations

Several limitations of our study should be considered when interpreting our findings.

The modest sample size, particularly of the TRS group (n = 47), and the low frequency of rare genotypes limited statistical power, especially for recessive models. Given the observed minor allele frequencies, particularly for *ITPA* rs1127354, substantially larger sample sizes (n > 1000) would be required to reliably detect moderate genetic effects, highlighting the limited power of the present study. The analysis was restricted to three candidate variants and did not capture broader genetic variation within the purine pathway. Missing genotype data further reduced the effective sample size in some analyses.

Significant age and smoking differences between cases and controls represent potential sources of confounding, although multivariable adjustment was constrained by sparse genotype strata. Control group minor allele frequencies for all three SNPs were consistent with gnomAD v4 European population reference data (Karczewski et al., 2020), suggesting that the control sample is broadly representative of the European population despite the age difference.

The absence of direct biochemical measurements of purine metabolites limits mechanistic interpretation and underscores the need for integrative genetic–metabolomic approaches.

Finally, the cross-sectional design precludes causal inference and does not capture longitudinal treatment trajectories.

## Future perspectives

Future studies should consider the interactions between genetic variability in purine metabolism and environmental modulators of adenosine signalling. Factors such as caffeine intake, smoking, and pharmacological modulation of purine metabolism may influence adenosine availability and receptor activity, potentially modifying clinical phenotypes and treatment response.

Cigarette smoking has been linked to changes in enzymes such as adenosine deaminase and ecto-5′-nucleotidase (CD73), thereby altering extracellular purine metabolism (Hu et al., 2025; Tian et al., 2021). Caffeine, a nonselective A1/A2A receptor antagonist, may act as a form of self-medication by transiently modifying adenosine receptor signalling, with particular relevance to negative symptoms and cognitive function. Conversely, xanthine oxidase inhibitors such as allopurinol can enhance adenosine signalling and have been explored as adjunctive treatments in schizophrenia (Lara et al., 2006; Pinheiro et al., 2022; Janho et al., 2026). Within this broader context, genetic variability in enzymes regulating purine metabolism, such as ATIC, AMPD1, and ITPA, may contribute to interindividual differences in adenosine tone and potentially modulate response to environmental or pharmacological influences. The present findings provide preliminary human genetic evidence that such variability may be clinically relevant in TRS subgroups. While the present findings do not directly address gene–environment interactions, they provide preliminary support for further investigation of adenosine-related pharmacogenetic mechanisms in TRS.

Future studies should also adopt a multi-omics approach integrating genetic profiling with adenosine and purine metabolomics to define biologically meaningful subgroups and to advance precision medicine approaches in schizophrenia.

Beyond the three candidate enzymes examined here, additional genes regulating purine metabolism and adenosine homeostasis — including adenosine deaminase (ADA), ecto-5′-nucleotidase (NT5E/CD73), and adenosine kinase (ADK) — have been implicated, directly or indirectly, in neuropsychiatric phenotypes and may further modulate adenosinergic tone in schizophrenia. Polymorphisms in adenosine receptor genes (ADORA1, ADORA2A) have also been associated with antipsychotic response and symptom dimensions in schizophrenia (Turčin et al., 2016).

An additional layer of complexity arises from the gut microbiota, which is increasingly recognized as a modulator of purine metabolism through gut–immune–inflammatory pathways. Certain commensal bacteria produce inosine, a purine nucleoside capable of interacting with adenosine receptors and thereby influencing host neuromodulatory and immune signalling (Tizabi et al., 2024). Given that alterations in gut microbiota composition have themselves been implicated in schizophrenia through the microbiota–gut–brain axis, it is plausible that microbial purine metabolism intersects with the host genetic variation examined in this study, potentially contributing to additional variability in adenosinergic tone not captured by genetic profiling alone. While this interaction remains speculative in the context of our current findings, it represents a compelling direction for future integrative research combining host pharmacogenetics, microbiome composition, and purine metabolomics.

## Conclusion

This study provides preliminary evidence that genetic variation within the adenosine metabolism pathway may contribute to clinical heterogeneity in schizophrenia. Specifically, *ATIC* rs2372536 was associated with TRS and showed exploratory associations with negative symptom severity, supporting a potential role of adenosinergic mechanisms in antipsychotic response. In contrast, *ITPA* rs1127354 demonstrated only a nominal association with schizophrenia risk that did not withstand correction for multiple testing and was based on very small genotype counts, while *AMPD1* rs17602729 showed no evidence of involvement in treatment resistance and only nominal associations with symptom burden.

These findings should be interpreted with caution and require replication in larger, independent cohorts before any clinical translation can be considered. Integration of genetic data with biochemical measures of purine and adenosine metabolism will be essential to establish biological relevance. Replication of the primary *ATIC* rs2372536–TRS association in adequately powered, multi-site cohorts represents the immediate priority for future work, and we intend to pursue this through collaborative European psychiatric genomics networks.

If validated, biomarkers within the adenosine pathway could contribute to biologically informed patient stratification and support the development of precision-oriented, mechanism-based therapeutic strategies.

## Data Availability

The datasets presented in this article are not publicly available because they contain sensitive individual-level genetic and psychiatric clinical information and unrestricted public deposition was not explicitly covered by the participant consent and/or ethics approval. Aggregate results and all non-sensitive supporting materials are provided in the article and Supplementary Material. Appropriately de-identified data supporting the manuscript will be made available to qualified researchers upon request. Requests to access the data should be directed to the Corresponding author (Milica Pjevac, milica.pjevac@psih-klinika.si). Requests will be evaluated by the authors and the responsible institution and/or ethics committee in accordance with the participant consent, the conditions of the ethical approval, and applicable data-protection requirements.
