# Supplementary material for: Genetic variants in purine metabolism and treatment resistance in schizophrenia: evidence supporting the adenosine hypothesis – a pilot study
Source: Front Pharmacol. 2026 Jul 28;17:1876365. doi: 10.3389/fphar.2026.1876365 (PMC13458008; doi:10.3389/fphar.2026.1876365)
Supplement: Supplementary file 1 [file Table1.docx]

**Supplementary Table S1:** Genotype distributions of *AMPD1* rs17602729, *ATIC* rs2372536, and *ITPA* rs1127354 in schizophrenia patients and healthy controls.

| **Gene / SNP** | **Genotype** | **Controls n (%), N = 93** | **Patients n (%), N = 249** | **χ²** | **p-value** |
| --- | --- | --- | --- | --- | --- |
| ***AMPD1* rs17602729** | **CC** | 69 (77.5%) | 184 (73.9%) | 0.68 | 0.710 |
|  | CT | 19 (21.3%) | 63 (25.3%) |  |  |
|  | TT | 1 (1.1%) | 2 (0.8%) |  |  |
| ***ATIC* rs2372536** | **CC** | 38 (41.3%) | 89 (38.7%) | 1.50 | 0.473 |
|  | CG | 37 (40.2%) | 108 (47.0%) |  |  |
|  | GG | 17 (18.5%) | 33 (14.3%) |  |  |
| ***ITPA* rs1127354** | **CC** | 74 (80.4%) | 203 (85.7%) | 7.01 | **0.030*** |
|  | CA | 14 (15.2%) | 33 (13.9%) |  |  |
|  | AA | 4 (4.3%) | 1 (0.4%) |  |  |

*Data are presented as counts (n) and percentages (%). Percentages are calculated based on the number of valid genotypes per SNP. Differences in genotype distributions between groups were assessed using χ² tests (3 × 2 contingency table). Missing genotypes per SNP - controls: AMPD1 n = 4, ATIC n = 1, ITPA n = 1; patients: AMPD1 n = 62, ATIC n = 19, ITPA n = 12. No associations remained significant after Bonferroni correction for multiple testing (α_adj = 0.0056). *p < 0.05 (nominal, uncorrected).*
