# Supplementary material for: Genetic variants in purine metabolism and treatment resistance in schizophrenia: evidence supporting the adenosine hypothesis – a pilot study
Source: Front Pharmacol. 2026 Jul 28;17:1876365. doi: 10.3389/fphar.2026.1876365 (PMC13458008; doi:10.3389/fphar.2026.1876365)
Supplement: Supplementary file 2 [file Table2.docx]

**Supplementary Table S2:** Genotype distributions of *AMPD1* rs17602729, *ATIC* rs2372536, and *ITPA* rs1127354 in treatment-resistant (TRS) and treatment-responsive patients with schizophrenia.

| **Gene / SNP** | **Genotype** | **TRS n (%)** | **Treatment-responsive n (%)** | **χ²** | **p-value** | **Missing (TRS / resp)** |
| --- | --- | --- | --- | --- | --- | --- |
| ***AMPD1* rs17602729** | **CC** | 34 (72.3%) | 150 (74.3%) | 1.29 | 0.526 | 2 / 9 |
|  | CT | 12 (25.5%) | 51 (25.2%) |  |  |  |
|  | TT | 1 (2.1%) | 1 (0.5%) |  |  |  |
| ***ATIC* rs2372536** | **CC** | 9 (25.7%) | 80 (41.0%) | 10.86 | **0.004**** | 12 / 7 |
|  | CG | 25 (71.4%) | 83 (42.6%) |  |  |  |
|  | GG | 1 (2.9%) | 32 (16.4%) |  |  |  |
| ***ITPA* rs1127354** | **CC** | 36 (87.8%) | 168 (85.3%) | 0.29 | 0.864 | 6 / 5 |
|  | CA | 5 (12.2%) | 28 (14.2%) |  |  |  |
|  | AA | 0 (0.0%) | 1 (0.5%) |  |  |  |

*Data are presented as counts (n) and percentages (%). Percentages are calculated based on the number of valid genotypes per SNP. TRS: treatment-resistant schizophrenia; resp: treatment-responsive. Differences in genotype distributions were assessed using χ² tests (3 × 2 contingency table). Missing genotype counts are listed per group (TRS / treatment-responsive). The effective sample size for ATIC rs2372536 was n = 35 (TRS) and n = 195 (responsive). Bonferroni-corrected significance threshold: α_adj = 0.0056 (9 tests: 3 SNPs × 3 genetic models). Green shading indicates significance after Bonferroni correction. *p < 0.05 (nominal); **p < 0.0056 (Bonferroni-corrected).*
