# Supplementary material for: Genetic variants in purine metabolism and treatment resistance in schizophrenia: evidence supporting the adenosine hypothesis – a pilot study
Source: Front Pharmacol. 2026 Jul 28;17:1876365. doi: 10.3389/fphar.2026.1876365 (PMC13458008; doi:10.3389/fphar.2026.1876365)
Supplement: Supplementary file 3 [file Table3.docx]

Supplementary Table S3: Logistic regression analyses of genetic variants in purine metabolism genes (*AMPD1* rs17602729, *ATIC* rs2372536, and *ITPA* rs1127354) and schizophrenia risk.

| **Gene / SNP** | **Model** | **n** | **Unadjusted OR (95% CI) p** |
| --- | --- | --- | --- |
| ***AMPD1* rs17602729** | **Additive** | 333 | 1.23 (0.65–2.35) p=0.526 |
|  | Dominant | 333 | 1.18 (0.59–2.36) p=0.647 |
|  | Recessive | 333 | 3.17 (0.28–35.65) p=0.351 |
| ***ATIC* rs2372536** | **Additive** | 316 | 1.03 (0.62–1.70) p=0.919 |
|  | Dominant | 316 | 1.97 (0.89–4.37) p=0.096 |
|  | Recessive | 316 | 0.15 (0.02–1.14) p=0.066 |
| ***ITPA* rs1127354** | **Additive** | 324 | 0.72 (0.29–1.79) p=0.478 |
|  | Dominant | 324 | 0.76 (0.28–2.06) p=0.594 |

*OR, odds ratio; CI, confidence interval. Reference genotype: major homozygote (CC) for all SNPs. Controls n = 93; patients n = 249 (n valid per SNP varies due to missing genotype data). Additive model: minor allele count (0/1/2). Dominant: any minor allele vs reference. Recessive: minor homozygote vs rest. No associations with schizophrenia risk were significant after Bonferroni correction (α = 0.0056). ITPA recessive model omitted (no patients with AA genotype in this comparison). *p < 0.05.*
